# SCANPatient: study protocol for a multi-centre, batched, stepped wedge, comparative effectiveness, randomised clinical trial of synoptic reporting of computerised tomography (CT) scans assessing cancers of the pancreas

**DOI:** 10.1186/s13063-024-08196-5

**Published:** 2024-06-17

**Authors:** Lin Li, Jessica Kasza, Ariadna Recasens, Liane Ioannou, Elysia Greenhill, Neil Merrett, David Cavallucci, Samantha Ellis, Helen Madgwick, Hyun Soo Ko, Lorraine Chantrill, Benjamin Loveday, Mehrdad Nikfarjam, Daniel Croagh, Jessica Yang, Andrew Dwyer, John Zalcberg, Charles Pilgrim, SCANPatient Authorship Group

**Affiliations:** 1 School of Public Health and Preventive Medicine, Monash University, Melbourne, VIC Australia; 2 Monash Program, Alfred Health, Melbourne, VIC Australia; 3 Department of Surgery, Western Sydney University, Sydney, NSW Australia; 4 Department of Surgery, Royal Brisbane and Women’s Hospital, Herston, QLD Australia; 5 Department of Radiology, Alfred Health, Melbourne, VIC Australia; 6 CRP Consumer Reference Group, Monash University, Melbourne, VIC Australia; 7 Department of Cancer Imaging, The Peter MacCallum Cancer Centre, Melbourne, VIC Australia; 8 The Sir Peter MacCallum Department of Oncology, The University of Melbourne, Melbourne, VIC Australia; 9 Department of Medical Oncology, Wollongong Hospital, Wollongong, NSW Australia; 10 Faculty of Science, Medicine and Health, University of Wollongong, Wollongong, NSW Australia; 11 Department of Surgery, Royal Melbourne Hospital, Parkville, VIC Australia; 12 Department of Surgery, Austin Health, Heidelberg, VIC Australia; 13 Department of Surgery, Monash Medical Centre, Melbourne, VIC Australia; 14 Department of Radiology, Concord Hospital, Concord, NSW Australia; 15 SA Node National Imaging Facility, Flinders Medical Centre, Bedford Park, SA Australia; 16 Cancer Research Program, School of Public Health and Preventive Medicine, Monash University, Level 5, 553 St Kilda Road, Melbourne, VIC 3004 Australia

**Keywords:** Pancreatic ductal adenocarcinoma (PDAC), Resectability, Computerised tomography (CT) scans, Synoptic reporting, Randomised controlled trial

## Abstract

**Background:**

Complete surgical removal of pancreatic ductal adenocarcinoma (PDAC) is central to all curative treatment approaches for this aggressive disease, yet this is only possible in patients technically amenable to resection. Hence, an accurate assessment of whether patients are suitable for surgery is of paramount importance. The SCANPatient trial aims to test whether implementing a structured synoptic radiological report results in increased institutional accuracy in defining surgical resectability of non-metastatic PDAC.

**Methods:**

SCANPatient is a batched, stepped wedge, comparative effectiveness, cluster randomised clinical trial. The trial will be conducted at 33 Australian hospitals all of which hold regular multi-disciplinary team meetings (MDMs) to discuss newly diagnosed patients with PDAC. Each site is required to manage a minimum of 20 patients per year (across all stages). Hospitals will be randomised to begin synoptic reporting within a batched, stepped wedge design. Initially all hospitals will continue to use their current reporting method; within each batch, after each 6-month period, a randomly selected group of hospitals will commence using the synoptic reports, until all hospitals are using synoptic reporting. Each hospital will provide data from patients who (i) are aged 18 or older; (ii) have suspected PDAC and have an abdominal CT scan, and (iii) are presented at a participating MDM. Non-metastatic patients will be documented as one of the following categories: (1) locally advanced and surgically unresectable; (2) borderline resectable; or (3) anatomically clearly resectable (Note: Metastatic disease is treated as a separate category). Data collection will last for 36 months in each batch, and a total of 2400 patients will be included.

**Discussion:**

Better classifying patients with non-metastatic PDAC as having tumours that are either clearly resectable, borderline or locally advanced and unresectable may improve patient outcomes by optimising care and treatment planning. The borderline resectable group are a small but important cohort in whom surgery with curative intent may be considered; however, inconsistencies with definitions and an understanding of resectability status means these patients are often incorrectly classified and hence overlooked for curative options.

**Trial registration:**

The SCANPatient trial was registered on 17th May 2023 in the Australian New Zealand Clinical Trials Registry (ANZCTR) (ACTRN12623000508673).

**Supplementary Information:**

The online version contains supplementary material available at 10.1186/s13063-024-08196-5.

## Administrative information

Note: the numbers in curly brackets in this protocol refer to SPIRIT checklist item numbers. The order of the items has been modified to group similar items (see http://www.equator-network.org/reporting-guidelines/spirit-2013-statement-defining-standard-protocol-items-for-clinical-trials/).

**Table:** 

Title {1}	SCANPatient: Study protocol for a multi-centre, batched, stepped wedge, comparative effectiveness, randomised clinical trial of synoptic reporting of computerised tomography (CT) scans assessing cancer of the pancreas.
Trial registration {2a and 2b}	The SCANPatient trial has been registered in the Australian New Zealand Clinical Trials Registry (ANZCTR) (ACTRN12623000508673).
Protocol version {3}	3 November 2023, Protocol V1.3.
Funding {4}	Funded by the Australian Government’s Medical Research Future Fund (MRFF) Rare Cancers, Rare Diseases and Unmet Need grant awarded to Monash University (MRF2015163).
Author details {5a}	^1^School of Public Health and Preventive Medicine, Monash University, Victoria, Australia. ^2^Monash Program, Alfred Health, Victoria ^3^Department of Surgery, Western Sydney University, NSW ^4^Department of Surgery, Royal Brisbane and Women’s Hospital, QLD ^5^Department of Radiology, Alfred Health, Victoria ^6^CRP Consumer Reference Group, Monash University, Victoria ^7^Department of Cancer Imaging, The Peter MacCallum Cancer Centre, Melbourne, Victoria ^8^The Sir Peter MacCallum Department of Oncology, The University of Melbourne, Victoria ^9^Department of Medical Oncology, Wollongong Hospital, NSW ^10^Faculty of Science, Medicine and Health, University of Wollongong, NSW ^11^Department of Surgery, Royal Melbourne Hospital, Victoria ^12^Department of Surgery, Austin health, Victoria ^13^Department of Surgery, Monash Medical Centre, Victoria ^14^Department of Radiology, Concord Hospital, NSW ^15^SA Node National Imaging Facility, Flinders Medical Centre, SA ^16^Department of Surgery, Alfred Health, Victoria, Australia.
Name and contact information for the trial sponsor {5b}	SCANPatient Research Fellow, Cancer Research Program, School of Public Health and Preventive Medicine Monash University Level 5, 553 St Kilda Road, Melbourne VIC 3004, Australia Tel: + 61 (3) 9903 0378 Email: SCANPatient@monash.edu
Role of sponsor {5c}	This is an investigator initiated clinical trial. The funder played no role in study design; collection, management, analysis, and interpretation of data; writing of the manuscript; and the decision to submit the manuscript for publication, and they will have no ultimate authority over any of these activities.

## Introduction

### Background and rationale {6a}

Approximately 4000 Australians are diagnosed with pancreatic cancers annually, over 80% of which are pancreatic ductal adenocarcinoma (PDAC) [1, 2]. Despite recent advances in treatment, overall survival in people with PDAC remains poor, with a 5-year survival across all stages of only 11% [2]. With no screening test for early detection of PDAC currently available [3], there is an urgent need to improve these outcomes particularly as it is predicted that PDAC will become the second most common cause of cancer death by the end of this decade [1–3]. Between half and two-thirds of patients present with metastatic disease at diagnosis [1–3]. Of those without obvious metastases at diagnosis, tumours are assessed as to their resectability according to the degree of major vascular involvement by the tumour [3]. Conventionally, major arterial resection has not been performed during pancreatic resections due to concerns about operative and oncologic risks. As such, non-metastatic disease may be categorised as (1) locally advanced (LA) and therefore initially unresectable without major vascular reconstruction; (2) borderline resectable (BR) due to some degree of vascular involvement; or (3) clearly resectable (CR) based on no major vascular involvement [3].

**Fig. 1:**
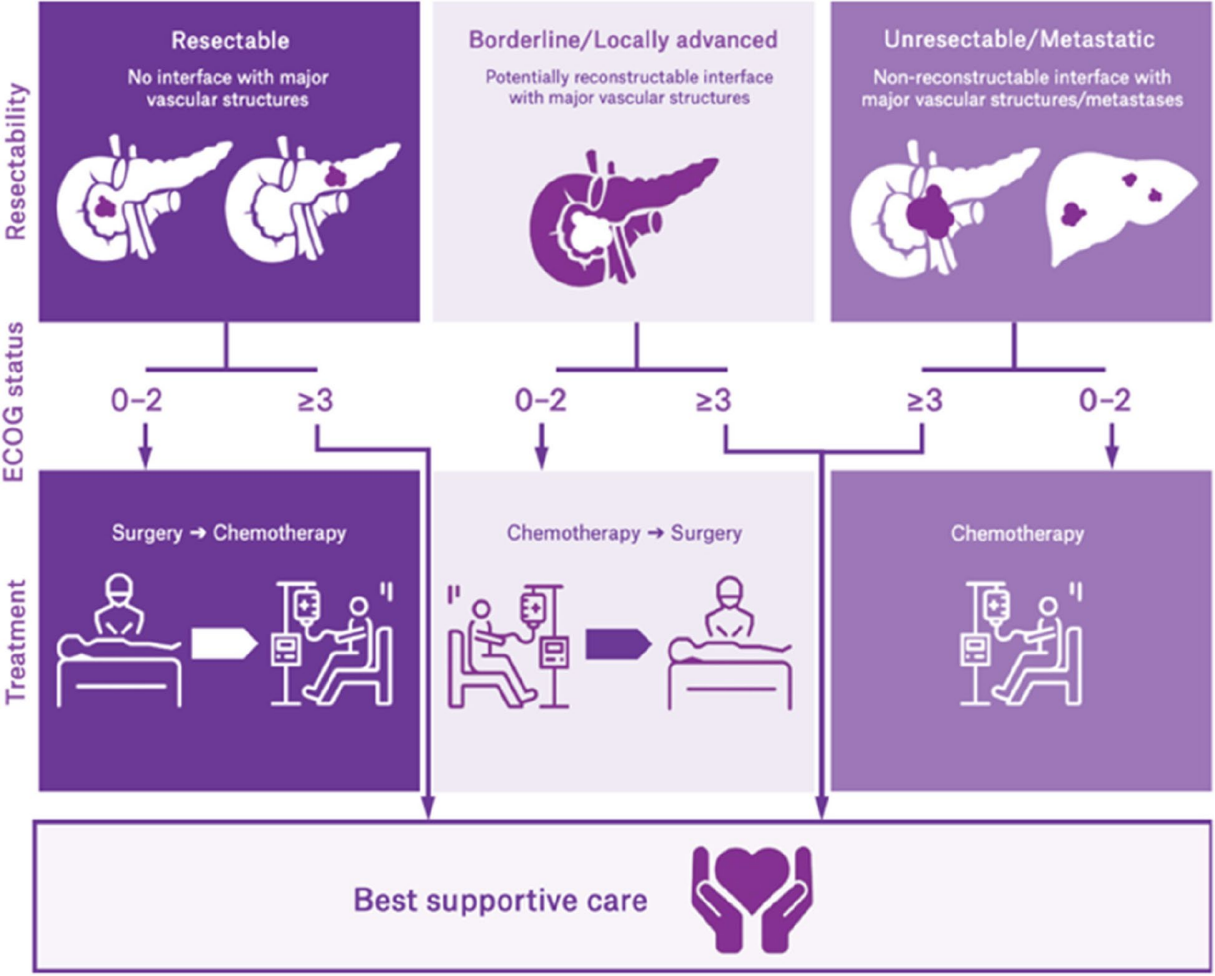
Stratification and treatment options for people diagnosed with pancreatic cancer

Stratification into LA, BR and CR which determines optimal treatment (Fig. 1) requires high-quality, cross-sectional radiological imaging using computed tomography (CT) using a dedicated “CT pancreas protocol” [3]. Magnetic resonance imaging (MRI) scans may also be used to help discriminate the extent of localised disease but CT is the current “gold” standard. The respective percentages of tumours in each of these categories (CR, BR or LA) in any given cohort around the world are uncertain [4], given differing published definitions of each subtype, as well as known variation in the interpretation of CT scans by radiologists and pancreatic surgeons [5]. For example, in radiologic staging, the following factors may contribute to variability: scanner (e.g. image quality, specific protocols or contrast timing); accuracy of findings (radiologist awareness and experience); documentation of findings (clarity and completeness); context (timing of scans vs cytologic diagnosis); and synthesis (determining status from finding) [6].

These issues have confounded interpretation of findings both within and across cohorts [7–11], which has led to inability to directly compare international treatment trials or ensure consistent recommendations between MDT meetings in different sites or jurisdictions. However, the use of a standard synoptic report may assist in reproducible consistency and validity [7–11], objectives this study aims to assess.

In an attempt to minimise variation in assessment of resectability of PDAC, our group conducted a small-scale pilot study in which we developed and tested a synoptic report for PDAC derived from the International Consensus Guidelines on Surgical Resectability [11] at two metropolitan pancreatic cancer services in Melbourne, Victoria [12]. The study found that the use of a synoptic report was feasible and had the potential to improve the classification of resectability [12]. Building on the experience and findings of the pilot study, we plan to trial the synoptic radiological report Australia-wide. We plan to use a comparative effectiveness (CE) randomised clinical trial (RCT) design to assess its utility against current standards of care to test whether this approach increases the proportion of PDAC categorised as BR, through structured assessment of vascular involvement.

### Objectives {7}

The primary objective of this trial is to determine the difference in the proportion of patients diagnosed with BR PDAC before and after introduction of the synoptic radiology report template. There are a number of secondary objectives. The description of objectives, together with the specific outcomes and timepoints are summarised in Table 1.

**Table 1:** Objectives, specific outcomes of the SCANPatient trial

Outcome	Rationale (description of primary and secondary objectives)	Source of data	Timepoint(s)
***Primary***			
Patients diagnosed with BR PDAC	To determine the change in the proportion of patients diagnosed with BR PDAC before and after introduction of the synoptic report	SCANPatient REDCap Database	At the time of each patient’s assessment
***Secondary***			
Patients diagnosed with CR PDAC	To determine the change in the proportion of patients with CR in a prospective cohort of patients with PDAC	SCANPatient REDCap Database	At the time of each patient’s assessment
Patients diagnosed with BR PDAC	To determine the change in the proportion of patients with BR in a prospective cohort of patients with PDAC	SCANPatient REDCap Database	At the time of each patient’s assessment
Patients diagnosed with LA PDAC	To determine the change in the proportion of patients with LA in a prospective cohort of patients with PDAC	SCANPatient REDCap Database	At the time of each patient’s assessment
Patients with CR PDAC planned to receive neoadjuvant chemotherapy	To determine the change in the proportion of patients with CR PDAC planned to have neoadjuvant chemotherapy	MDT summary	At the time of the MDT
Patients with BR PDAC planned to receive neoadjuvant chemotherapy	To determine the change in the proportion of patients with BR PDAC planned to have neoadjuvant chemotherapy	MDT summary	At the time of the MDT
Patients with LA PDAC planned to receive neoadjuvant chemotherapy	To determine the change in the proportion of patients with LA PDAC planned to have neoadjuvant chemotherapy	MDT summary	At the time of the MDT
Patients undergoing R1 resection	To determine the change in the proportion of patients undergoing R1 resection (microscopic margin positivity)	SCANPatient REDCap Database	At the time of surgery (if undertaken)
Patients undergoing R2 resection	To determine the change in the proportion of patients undergoing R2 resection (macroscopic margin positivity)	SCANPatient REDCap Database	At the time of surgery (if undertaken)
Patients surgery is abandoned	To determine the change in the proportion of patients whose surgery is abandoned intraoperatively	Surgical operation report	At the time of surgery (if undertaken)
Patient’s management strategy changed	To determine the change in the management strategies for patients with CR, BR and LA PDAC	MDT summary	At time of MDT
Patients with CR PDAC survival status	To determine the overall survival rate for patients with CR localised PDAC	Sites and the national death registry	6 months post completion of recruitment at end of trial
Patients with BR PDAC survival status	To determine the overall survival rate for patients with BR localised PDAC	Sites and the national death registry	6 months post completion of recruitment at end of trial
Patients with LA PDAC survival status,	To determine the overall survival rate for patients with LA localised PDAC	Sites and the national death registry	6 months post completion of recruitment at end of trial
Satisfaction of specialists with synoptic report	To determine the satisfaction of radiologists and HPB (hepatobiliary) surgeons involved in this trial	Health service survey	Prior to the introduction of synoptic reporting and 6 months post commencing synoptic reporting
Synoptic report used routinely as standard of care	To determine the extent to which the synoptic reports for PDAC has become standard of care in routine MDT meetings and whether this model has been incorporated into in-house RIS/PACS (radiology information systems and picture archiving and communication systems)	Health service survey	6 months after trial complete

We hypothesise that the introduction of the synoptic radiological template report will alter (increase) the rate of diagnosis of BR PDAC from baseline.

### Trial design {8}

This is a batched stepped wedge, comparative effectiveness, cluster randomised trial [13]. Participating sites will start the study in three batches; data collection in each batch starts 2 months after the start of the previous batch. Note that study power is unaffected by the exact timing of the start of each successive batch [13]. In each batch, hospitals will be randomised to the sequences of a five-sequence stepped wedge design, as indicated in Table 2. Initially all hospitals will continue to use their current standard radiology reporting (the comparator) for a period of between 6 and 30 months. In each batch, after each 6-month period, a randomly allocated group of hospitals will commence using the synoptic report template (the intervention), until all hospitals are using the synoptic report.

**Table 2:**
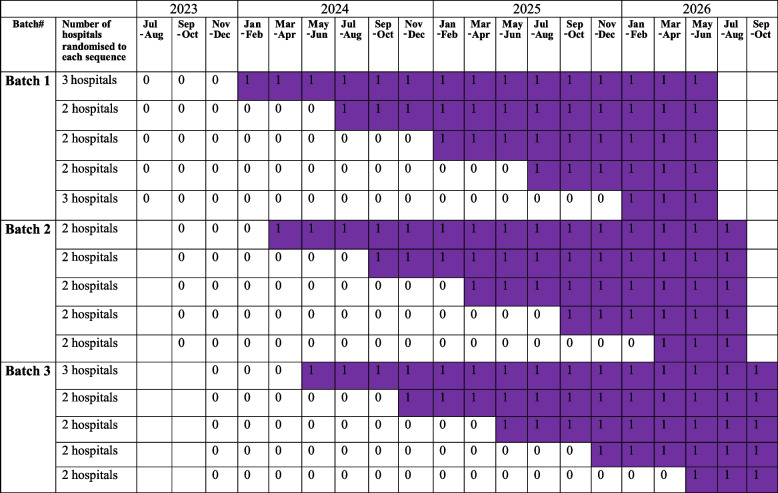
Batched stepped wedge cluster randomised design—standard (represented as “0”) vs Synoptic Reports (represented as “1” in purple)

## Methods: participants, interventions, and outcomes

### Study setting {9}

The trial will be based at 33 Australian hospitals. A list of participating hospitals can be found via the following weblink in the ANZCTR: https://www.anzctr.org.au/ACTRN12623000508673.aspx.

Participating sites will provide data from eligible patients over the 36-month data collection period, with the first batch’s data collection period starting from July 2023.

### Eligibility criteria {10}

#### Inclusion criteria

The unit of randomisation here is the hospital. Hospitals are randomised at the time they commence synoptic reporting. The hospitals participating in the study must satisfy the following requirements:

- Hold a multi-disciplinary team meeting (MDM) where PDAC cases are presented;
- Are prepared for radiology reporting methods to be randomised;
- Manage on average ≥ 20 patients with PDAC annually (across all stages), and
- Are not currently using a synoptic radiological report for pancreatic cancer.

Each participating hospital will be providing access to relevant information of their patients with pancreatic cancer. Once presented to an MDM, follow-up data will be collected on all patients presented at the MDM, provided the patients are:

1. Aged 18 years of age or older;
2. Have suspected or proven adenocarcinoma of the pancreas; and
3. Have an abdominal CT scan (either performed by hospital radiology departments or an external radiology service) as a part of standard clinical care for diagnostic purposes

#### Exclusion criteria

Patients with neuroendocrine tumours will be excluded from the study. No inclusion waivers will be allowed.

### Who will take informed consent? {26a}

Not applicable. Individual patient consent is not required. A waiver of consent has been granted by the Monash Health Human Research Ethics Committee (HREC).

### Additional consent provisions for collection and use of participant data and biological specimens {26b}

Not applicable. A waiver of consent has been granted by the Monash Health HREC. No biological specimens will be used*.*

## Interventions

### Explanation for the choice of comparators {6b}

#### Control group

The comparator for this study is continued use of the baseline standard radiology reporting process (i.e. standard reporting) at each of the participating sites (prior to the commencement of synoptic reporting).

Current standard radiology reporting varies, with the level of detail and the style of the report being radiologist dependent. The report is typically dictated by radiologists reviewing scans and usually written as a narrative of varying lengths and complexity, finishing with a conclusion. We will accurately document the current standard reporting process, and later compare it with the synoptic reporting template that is under study.

### Intervention description {11a}

#### Experimental group

After a site commences the use of synoptic radiology reporting (i.e. synoptic reporting), the site radiologist reviewing CT scans will use a structured reporting tool that was developed in the pilot study [12] to describe the patient’s pancreatic lesion in detail. The synoptic report template collects 63 discrete fields of data that concisely describe the pancreatic cancer mass characteristics, blood vessel, and other adjacent structure involvement, and determines the extent of local disease spread. The radiologist will assess a patient’s resectability status (based on the international consensus guidelines) by completing the synoptic report in a Research Electronic Data Capture (REDCap) application. The synoptic reporting template has been previously described [12]. The median time needed to complete a synoptic report for a patient by a radiologist during the pilot study was 4 min, so it is assumed the time will be similar for this study.

### Criteria for discontinuing or modifying allocated interventions {11b}

Not applicable. As mentioned above, after a site commences synoptic reporting, all the site radiologist/s will be reporting the CT scans using synoptic reporting rather than standard reporting. For trial patients, there is no “intervention” as such that any of them would need to discontinue.

### Strategies to improve adherence to interventions {11c}

Training will be provided to the sites’ radiologists by a project data manager and an experienced radiologist who has previously used the synoptic reporting tool. The training session will be delivered online and it will take about 30 min to complete. Training will commence 1 month prior to the start of the synoptic reporting phase of a sites’ participation.

To monitor adherence to the synoptic reporting tool, the data manager in the central project team will ensure data completeness and accuracy by undertaking quality assurance checks and ensuring ongoing training and support to participating radiologists is available.

### Relevant concomitant care permitted or prohibited during the trial {11d}

Not relevant, this study relates only to the discussion and planning phase of treatment and there will be no impact on concomitant or future care subsequent to the MDM presentation.

### Provisions for post-trial care {30}

Not relevant. This study relates only to the discussion and planning phase of treatment.

### Outcomes {12}

The primary outcome is the proportion of patients diagnosed with BR PDAC, aiming to detect a change in this proportion before and after introduction of the synoptic report. There are a number of secondary outcomes (see Table 1).

### Participant timeline {13}

**Fig. 2:**
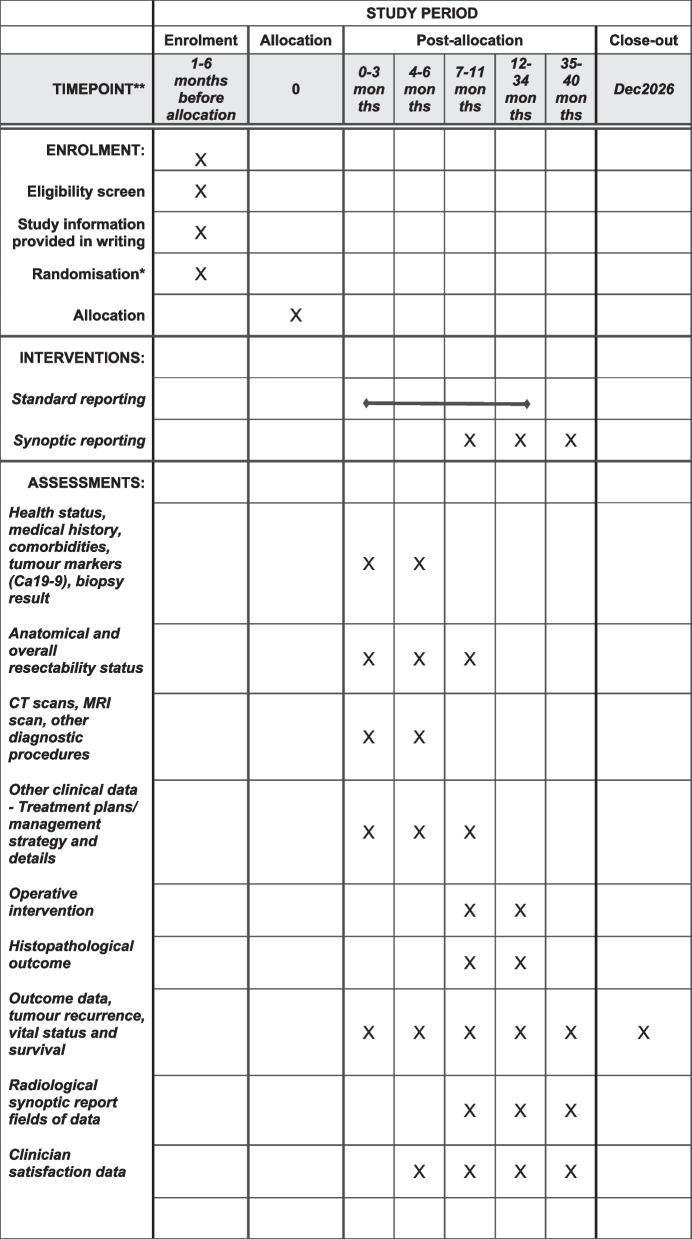
SPIRIT figure—Schedule of enrolment, interventions, and assessments of SCANPatient clinical trial**.** This is a batched, stepped wedge cluster randomised trial, more details of randomisation and sequencing can be found in Table 2. **For more details about timepoints for hospital allocation, please see Table 2

After obtaining site governance authorisation, randomisation, and sequence allocation (Fig. 2), all patients who undergo presentation of radiological staging of PDAC at each participating institution MDM will be included in the study, and their baseline data and subsequent data will be collected as per the descriptions in Table 3.

**Table 3:** Data required, sources, methods and time to collect them^a^

What data and information are needed	Main sources and methods to collect/extract data	When to collect (Timepoint)		
		Baseline (diagnostic phase)^b^	Post-operative	12 and 24 months
Patient demographics, i.e. given name^c^, surname^c^, date of birth^c^, age, sex	MDM Report and radiology report, provided by participating radiologists or site focal point of contacts; extracted and enter into REDCap database by SCANPatient Project team	**✔**		
Health status, medical history, comorbidities, tumour markers (Ca19-9)	As above	**✔**		
Anatomical and overall resectability status (i.e. clearly resectable (CR), borderline resectable (BR), locally advanced (LA), or metastatic cancer)	As above	**✔**	**✔**	
CT scans, MRI scan, other diagnostic procedures *(As repeated per patient)*	As above	**✔**		
Other clinical data—Treatment plans/ management strategy and details, e.g. use of chemotherapy, radiology, best supportive care, and other forms of ca treatment	As above	**✔**	**✔**	
Operative intervention (surgical resection details, whether completed or abandoned, blood loss and other relevant operation details)	Surgical operation report, provided by participating surgeons, or site contacts; extracted by SCANPatient Project team		**✔**	
Histopathological outcome (resected tumour details, surgical resection margin assessment, lymph nodes, TNM staging, and other relevant histopathological details)	Pathology report of operation specimen, provided by participating surgeons, or site contacts; extracted by SCANPatient Project team		**✔**	
Outcome data, tumour recurrence, vital status (i.e. alive, dead, unknown, or lost to follow-up) and survival	As above; Data linkage to allow assessment of overall survival: link to AIHW Death Registry or to individual state death registries at the end of study; other possible death checks	**✔**	**✔**	**✔**
Radiological synoptic report fields of data^d^ (CT scan focused, after switching to synoptic reporting) • Quality assurance characteristics • Basic details • Distant metastases • Mass characteristics • Arterial evaluation • Venous evaluation • Conclusion and resectability status	Complete online (via central REDCap database) by radiologists from those sites that have been randomised to synoptic reporting		**✔**	**✔**
Clinician satisfaction data	Participating radiologists and hepatobiliary surgeons will be asked to complete an online survey using REDCap or Qualtrics at two time points to assess clinician satisfaction. The survey is developed by the project team (see Appendix 1). Responses to the survey are anonymised		**(**The first timepoint to assess clinician satisfaction is prior to site randomisation to synoptic reporting)	**(**The second timepoint is 6 months after randomisation to synoptic reporting)

*MDM* multi-disciplinary team meeting

^a^No additional investigations beyond standard care or follow-up information outside of routine audit will be required or collected

^b^Some data present at baseline will be collected over the ensuing 3–6 months from health records

^c^Identifiable data per site approval

^d^There are around 60 discrete fields of data in the synoptic reporting template

### Sample size {14}

Currently 8% of all patients diagnosed with PDAC are categorised with BR disease in the Upper Gastrointestinal Cancer Registry (UGICR; unpublished data) which is operating in most major centres in Victoria and approximately half of the major centres in New South Wales. We hypothesise that with the introduction of a new structured radiology report, leading to increased diagnostic accuracy, this will increase to 15%. This is supported by the final results of the pilot synoptic report study (wherein the rate of BR disease was 13% in the pilot study across 95 patients [12]) and international registry data which have shown that the rate of resection of major vascular structures at the time of surgery for PDAC is between 10 and 15% [14], a surrogate for involvement of those major structures and therefore a pseudo-definition of BR disease. As not all patients with BR PDAC will require formal venous or arterial resection, it is likely the actual rate of BR disease is higher in these larger international and national registries. An increase in the diagnosed rate of BR disease to this level will result in more patients appropriately being given neoadjuvant therapy, and a clinically meaningful change in the standard of care if identified.

The trial was originally designed as a standard, stepped wedge design with 8 hospitals randomised to each sequence of a standard 5-sequence/6-period stepped wedge design (a total of 40 hospitals). With 15 patients per hospital in each 6-month period, assuming a two-sided significance level of 0.05, this design has power ≥ 80% to detect an increase from 8 to 15% in the primary outcome. Power is maintained for a wide range of intracluster correlations and alternative within-cluster correlation structures, e.g. for a discrete time decay within-cluster correlation structure [15], with an intracluster correlation of 0.1 and a cluster autocorrelation of 0.55, power is 84%. Under most within-cluster correlation structure assumptions, power will remain > 76% should hospital recruitment be up to 25% lower and patient recruitment up to 30% lower than expected (i.e. with 10 patients each year in each of 30 hospitals). With the batched variant shown in Table 2, for the extreme scenario considered above (an intracluster correlation of 0.1 and cluster autocorrelation of 0.55), power levels remain > 80%. Sample size calculations were performed using the Shiny CRT calculator [16].

### Recruitment {15}

Hospitals were invited to register their interest in participating in this trial via an Expression of Interest (EOI) process. An EOI form was initially distributed through professional networks and organisations such as the Australian & New Zealand Hepatic, Pancreatic and Biliary Association (ANZHPBA), the Abdominal Radiology Group Australia and New Zealand (ARGANZ) of the Royal Australian and New Zealand College of Radiologists (RANZCR), the Australasian Gastro-intestinal Trial Group (AGITG), and the Royal Australasian College of Surgeons (RACS). Eligible hospitals that submitted an EOI and agreed to participate were further contacted with the help and coordination from local surgeons and/or radiologists (many of whom acted as site PIs or AIs). Relevant ethics and research governance applications were prepared and submitted, and after the site governance authorisation has been granted for the trial to be conducted at a specific site, all patients who undergo radiological staging of PDAC at each participating hospital will be included in the study. Individual clinicians will list patients for MDM discussion at the participating sites as per usual care.

## Assignment of interventions: allocation

### Sequence generation {16a}

Hospitals will start the study in three batches. In each batch, hospitals will be randomised to one of the sequences of the stepped wedge design, as illustrated in Table 2. Randomisation is via computer-generated random numbers generated by the trial statistician. This sequence was sent to a researcher otherwise uninvolved with the trial.

### Concealment mechanism {16b}

The otherwise uninvolved researcher created ID numbers for each hospital, and the trial statistician was unaware of the link between ID numbers and hospitals. This uninvolved researcher then sent the sequence of hospitals to the first author. Hospitals will be informed that they will be switching to synoptic reporting about 1 month prior to their allocated switch time.

### Implementation {16c}

The trial statistician generated the allocation sequence. Since this is a cluster randomized trial with a waiver of consent, individual patient recruitment is not taking place.

## Assignment of interventions: blinding

### Who will be blinded {17a}

Only the first author and another two members of the central project team who run the trial on a daily basis will be aware of when each site is crossing over to synoptic reporting. Hospitals will be aware what reporting method they are implementing. All other chief investigators and data collectors will be blinded as to when they are due to commence synoptic reporting. They will not be part of the process in which relevant personnel will develop the randomisation sequences for various batches of trial sites, and the randomisation schedule will be kept confidential from them. Data analysts will be informed of the planned time of switching of hospitals, but will not be informed of hospital names.

### Procedure for unblinding if needed {17b}

Hospitals will be aware when they have been switched over to the synoptic reporting, so no procedure for unblinding is necessary.

## Data collection and management

### Plans for assessment and collection of outcomes {18a}

Working together with the site clinicians/key contacts, the SCANPatient Central team will collect/extract baseline, post-operative, and survival data from various sources, including the MDM summaries, CT scan report, operation report, and pathology report. This will provide details of all treatment including surgery.

The main data required, sources, methods, and timepoints to collect them are summarised in Table 3.

The satisfaction of radiologists and hepatopancreaticobiliary (HPB) surgeons involved in the SCANPatient trial with respect to their use of standard reporting and synoptic reporting will be assessed using an online Clinician Satisfaction Survey developed by the SCANPatient research team (see Appendix 1).

### Plans to promote participant retention and complete follow-up {18b}

The SCANPatient central team will work closely with the site teams to make the processes as easy and smooth as possible. Central team plans to (1) follow up periodically to make sure that sites are sending documents as planned; (2) create detailed instructions and training materials for sites to use relevant platforms; (3) communicate with IT of the sites to allow them to enable access; and (4) provide standard templates of annual reports for the site to use, etc.

### Data management {19}

The SCANPatient Project team will extract required data from the main sources and enter them into the study REDCap database. During the synoptic report period, radiologists at each participating site will complete the synoptic report and enter relevant information directly into a module of the central REDCap database. Relevant data from the sites will be securely transferred to the central team via MDrive or REDCap. The data manager will regularly create reports to check data completeness and quality, follow up sites for missing data, and undertake data quality checks. All data will be stored using encrypted, secured, and regulatory-approved platforms and methods.

### Confidentiality {27}

There is an adequate plan to protect the confidentiality of data. Measures and safeguards we take to ensure the confidentiality and privacy of data are included in the SCANPatient data management plans, for both identifiable data (Appendix 2) and de-identifiable data (Appendix 3). To maintain the confidentiality of the data, access to identifiable information will be restricted to the minimum needed number of people in the project team and participating site clinicians. The REDCap software to be used for data collection allows for differing user access rights and will be set up so that clinicians can only view patient information from their own site. Personal medical information will always be treated as confidential, according to local privacy laws.

The trial will be conducted in compliance with the protocol; good clinical practice guidelines; university research procedures; relevant Commonwealth and State-based legislations and principles governing privacy and confidentiality. All information will be handled in accordance with Australian or equivalent privacy laws.

### Plans for collection, laboratory evaluation, and storage of biological specimens for genetic or molecular analysis in this trial/future use {33}

N/A. No such biological specimens will be collected in this trial.

## Statistical methods

### Statistical methods for primary and secondary outcomes {20a}

Results will be reported according to the CONSORT extension for stepped wedge trials [17]. Baseline data will be presented in a tabular form with mean and standard deviation or median with lowest and highest values, or percentages and counts as appropriate, by treatment group. We will also present baseline data by hospital and time period.

Results will be compared between *standard reporting* and *synoptic reporting* phases. All available primary and secondary outcomes will be analysed at the patient level using mixed-effects regression models with random intercepts for each cluster in each period, assuming a discrete time decay within-cluster correlation structure (where possible), a fixed effects for the intervention and separate fixed effects for period within each batch [15], or binary outcomes, log-binomial models will initially be fitted; should these fail to converge, mixed-effects Poisson regression models will be fitted, with robust variance estimation. Results will be presented as relative risks with 95% confidence intervals, and as risk differences with 95% confidence intervals. The sensitivity of results to the assumed within-cluster correlation structure will be assessed by fitting models with random effects for each cluster only, and for each cluster-period within cluster. Estimated intracluster correlations and cluster autocorrelations will be reported. The survival outcome will be analysed using a Cox proportional hazards model including shared frailty terms for cluster. All tests will be two-sided. Analyses will be conducted in Stata v 17 or later or in R version 4.2.1 or later as appropriate.

A statistical analysis plan will be prepared and finalised prior to the dataset being released to the statistician.

### Interim analyses {21b}

No interim analyses will be conducted.

### Methods for additional analyses (e.g. subgroup analyses) {20b}

To assess whether there is evidence of the effect of synoptic reports changing over time, an additional analysis including an interaction between the intervention term and time since synoptic reports were introduced in each site will be conducted for the primary outcome.

### Methods in analysis to handle protocol non-adherence and any statistical methods to handle missing data {20c}

Given that the proportion of missing data for the primary outcome is expected to be small, the main sensitivity analysis for missing data on the primary outcome will involve imputing data under two scenarios: “worst-best” and “best–worst” scenarios. In the “worst-best” scenario, missing data for diagnosis in the intervention arm will be imputed as “not borderline resectable”, while missing data in the control arm will be imputed as “borderline resectable”. In the “best–worst” scenario, the imputation will be reversed. The primary outcome will be analysed as described above under each of these scenarios. If these two scenarios lead to different conclusions regarding the treatment effect, a multiple imputation approach to dealing with missing data for the primary outcome will be taken.

### Plans to give access to the full protocol, participant-level data, and statistical code {31c}

The datasets used and/or analysed during the current study can be made available by the corresponding author upon request and in agreement with the research collaboration and data transfer guidelines of Monash University.

## Oversight and monitoring

### Composition of the coordinating centre and trial steering committee {5d}

A Trial Management Committee (TMC) and three subcommittees (an Operations Subcommittee, a Clinical Reference Group and a Consumer Reference Group) have been formed to oversee various aspects of the study. These project teams meet regularly to discuss various issues and monitor study progress, and any technical or safety or compliance issues that may jeopardise the completion or integrity of the study. They ensure that project reporting and milestones are met in a timely manner.

Details of the composition, roles, and meeting frequency of each of SCANPatient trial committees/subcommittees are summarised in Table 4.

**Table 4:** The composition, roles and meeting frequency of SCANPatient trial committees and subcommittees

Name of committee/subcommittee	Composition	Main roles and responsibilities	Meeting frequency	Additional notes
Trial Management Committee (TMC)	The TMC is made up of all CIs and AIs, as well as a consumer representative	The role of the TMC is to oversee all aspects of the study (the operational elements of which will be delegated to the Trial Operations Sub Committee—see below) including liaison with partner organisations, risk management, training in the use of the synoptic tool and associated database, site recruitment including key link staff at each institution, management of the study throughout the recruitment period and subsequent analysis and reporting	The TMC meets at least quarterly in the first 12 months to ensure appropriate set-up processes are in place, and thereafter at a minimum of every 6 months unless required to meet more often	A Trial Management Committee (TMC) was formed at the outset of the grant (2021)
Trial Operations Subcommittee	It consists of all centrally employed personnel appointed to the study (including SCANPatient Fellow&Senior Research Officer&Data Manager), and the Upper Gastrointestinal Cancer Registry Program Manager, and CIA, CIJ, CII	In addition to the operational responsibilities referred to above, its role will be to prepare a formal protocol, obtain Ethics and Governance approval at the lead sites (Monash Health and Monash University) as well as at other sites as required, establish a central REDCap database accessible to all participating sites for entry of synoptic reports, site and central data management; and in conjunction with the statistical team develop electronic case record forms (eCRFs) for collection of short and longer term clinical data including baseline demographics as well as treatment and outcome data	It meets fortnightly at the outset, and more often when required	It was formed in August 2022
Consumer Reference Group	It is an eight-member group chaired by a consumer and attended by representatives of two pancreatic cancer patient groups—PANCARE and PANKIND, with additional representation from the AGITG (Australasian Gastrointestinal Trials Group) community advisory panel	The consumer reference group considers the communications strategy from a consumer perspective. The group has helped develop an appropriate ethics and governance framework, the trial opening announcement and, depending on results, will consider communication around how best to mainstream practices across the country	It meets at least yearly formally and more frequently when needed	It was formed in September 2022
Clinical Reference Group	This reference group is chaired by CIA and CIB and consists of 12 radiologists, surgeons and medical oncologists	The clinical reference group which takes responsibility for fine-tuning any of the details of the synoptic reporting and/or logistics from a radiological and surgical perspective as well as the implementation of these reports into day-to-day practice and the satisfaction survey This reference group will develop the tools to assess the satisfaction of clinicians regarding the implementation and use of the synoptic report, as well as some related substudies	It meets at least quarterly in the first 12 months, and thereafter at a minimum of every 6 months unless required to meet more often	It was formed in August 2022 This reference group helped develop the Clinician Satisfaction survey (see Appendix 1)

### Composition of the data monitoring committee, its role and reporting structure {21a}

N/A. No formal data monitoring committee has been established.

### Adverse event reporting and harms {22}

Patients in this trial are not having their care pathway altered in any way. The trial aims to improve the documentation of the CT scan review by using the synoptic report method which will not result in adverse events.

### Frequency and plans for auditing trial conduct {23}

The Sponsor may audit the investigational site to compare raw data, source data, and associated records with the interim (if applicable) or final report of the trial to assure that data have been accurately reported.

### Plans for communicating important protocol amendments to relevant parties (e.g. trial participants, ethical committees) {25}

After each important protocol amendment has been made and approved, we will inform relevant parties of the changes, using various channels of communication.

As SCANPatient is a multi-site study, after a protocol amendment has been approved by the reviewing HREC (Monash Health HREC), we will inform the Principal Investigators at all sites, and forward the approval letter and relevant correspondence and documents to the site research teams and Research Governance Offices (RGOs). Members of the TMC and relevant subcommittees will also be informed, and relevant changes will also be reflected into the ANZCTR registry.

### Dissemination plans {31a}

In addition to the ANZCTR registry and the funder (NHMRC-MRFF), progress and results will be shared with relevant audiences at appropriate times including conference abstracts/presentations, websites/newsletters of the SCANPatient project or collaborators/partners, talks at professional networks, reports and publications, etc. Data/results will only be reported in an aggregate form and every effort will be made to ensure individuals are not re-identifiable in research presentations/reports/publications.

Authorship of publications resulting from this trial will be based on the guidelines on authorship, such as those described in the Uniform Requirements for Manuscripts Submitted to Biomedical Journals. Named authors must (a) interpret the data; (b) provide critical review of the paper; and (c) give final approval of the final version.

The publications will recognise the contribution of investigators and, where journal and space allow, the names of all investigators will be listed in the Acknowledgements section.

## Discussion

Better classifying patients with non-metastatic PDAC as having tumours that are either clearly resectable, borderline resectable, or locally advanced and unresectable may improve patient outcomes by avoiding ineffective surgery, optimising care and treatment planning. The borderline resectable group are a small but important cohort in whom surgery with curative intent may be considered; however, inconsistencies with definitions and an understanding of resectability status means these patients are often incorrectly classified and hence overlooked for curative options.

The SCANPatient trial has built on a small-scale pilot study [12] and is now expanding the synoptic reporting approach to over 30 major hospitals across Australia. As the requirements and procedures of research ethics and governance applications (as well as the research management systems) in different states/jurisdictions and types of hospitals vary (e.g. public vs private hospitals, health services with or without radiological/MDM capacity), this multi-centred and stepped wedge clinical trial has its challenges in implementation. After some delays in site governance approvals, data collection finally started from early July 2023.

## Trial status

The most updated protocol version is Protocol V1.3, 3 November 2023. Recruitment started in July 2023 in the first batch of 12 trial sites. The second batch then started in the 10 sites in September, and the third and final batch of 11 sites in November of 2023. Recruitment will be completed in about September 2026.

### Supplementary Information

Supplementary Material 1.

## Data Availability

The study statistician and the central research team will have access to the final trial data. Site research teams will have access to the data collected from their own sites.

## Abbreviations

AGITG: Australasian Gastrointestinal Trial Group
AI: Associate investigator
ANZCTR: Australian New Zealand Clinical Trials Registry
ANZHPBA: Australian & New Zealand Hepatic, Pancreatic and Biliary Association
ARGANZ: Abdominal Radiology Group Australia and New Zealand
BR: Borderline resectable
CE: Comparative effectiveness
CR: Clearly resectable
CT: Computerised tomography
EOI: Expression of Interest
HPB: Hepatopancreaticobiliary
HREC: Human Research Ethics Committee
LA: Locally advanced
MDT: Multi-disciplinary team
MDM: Multi-disciplinary team meeting
MRFF: Medical Research Future Fund
MRI: Magnetic Resonance Imaging
NHMRC: National Health and Medical Research Council
PDAC: Pancreatic ductal adenocarcinoma
PI: Principal investigator
RACS: Royal Australasian College of Surgeons
RANZCR: Royal Australian and New Zealand College of Radiologists
RCT: Randomised clinical trial
REDCap: Research Electronic Data Capture
RGO: Research Governance Offices
SCANPatient: Synoptic reporting of Computerised tomography scans Assessing caNcer of the pancreas
TMC: Trial Management Committee
UGICR: Upper Gastrointestinal Cancer Registry
