# Supplementary material for: SCANPatient: study protocol for a multi-centre, batched, stepped wedge, comparative effectiveness, randomised clinical trial of synoptic reporting of computerised tomography (CT) scans assessing cancers of the pancreas
Source: Trials. 2024 Jun 17;25:388. doi: 10.1186/s13063-024-08196-5 (PMC11181632; doi:10.1186/s13063-024-08196-5)
Supplement: Supplementary file 1 — Supplementary Material 1. [file 13063_2024_8196_MOESM1_ESM.docx]

**Title**

**SCANPatient: Study protocol for a multi-centre, batched, stepped wedge, comparative effectiveness, randomised clinical trial of synoptic reporting of computerised tomography (CT) scans assessing cancers of the pancreas.**

**List of Appendices**

Appendix 1. Clinician Satisfaction Survey

Appendix 2. Data management plan – identifiable data

Appendix 3. Data management plan – de-identifiable data

Appendix 1. Clinician Satisfaction Survey

**SCANPatient Clinician Satisfaction Survey**

*[A note for Monash Health HREC: Please be advised this is an example of what we wish to use rather than the final version. Subsequently if there are changes we will inform the HREC.]*

The Clinician Satisfaction Survey is part of the MRFF funded SCANPatient trial, which aims to determine whether the introduction of a structured synoptic radiology reporting tool across institutions that manage pancreatic cancer provides greater accuracy in the diagnosis of resectable pancreatic compared to the current standard radiological approach for assessment of pancreatic CT scans. For a detailed description of the study, please visit this link or read this one-page document: Scanpatient project description

The objective of this survey is to determine the satisfaction of radiologists and hepatobiliary (HPB) surgeons involved in the SCANPatient trial in their use of the current standard reporting and synoptic reporting of CT scans of patients with pancreas cancer.

Your valuable input is essential to help us better understand your needs and preferences. This survey should take no more than 5 minutes to complete, and your responses will remain completely confidential.

**CONSENT**

By submitting the questionnaire form, you are confirming that you have read the description of the study and that you agree to participate in this survey.

**SCANPatient Clinician Satisfaction Survey – Prior to introduction of synoptic report**

1. What is your craft group?
2. Surgeon

a.1 What is your specialty?

- General surgery
- HPB surgery
- OG surgery
- Other specialty (free text)

b. Radiologistb.2 What is your specialty?

- General radiology

- Abdominal Imaging

- Other specialty (free text)

2. How many years of specialist practice do you have?

- a. 0 to <5
- b. 5 to <10
- c. ≥10

There are several guidelines that provide standardised criteria for the assessment of the resectabilitiy of pancreatic tumours. These are generally based on factors such as tumour size and vascular involvement to guide treatment decisions.

3. What system/s for classification of resectability is/are currently used in your MDT to describe cases of non-metastatic pancreas cancer? (If you participate in multiple MDMs, please respond to this and subsequent questions based on the primary/principal site where you practice). Feel free to tick more than one option.

- NCCN
- AGITG
- International consensus 2017
- Alliance A021101
- AHPBA/SSO/SSAT (2009)
- MD Anderson Cancer Centre (2006)
- No formal system
- Other (free text)

4. Is the primary/principal MDM you attend located in...

a. Public

b. Private

**Below are the questions that will appear if the respondent is a surgeon:**

5. On a scale of 1-5, how satisfied are you currently with the level of anatomical detail *DISCUSSED* in your MDM for CT scans of with pancreas cancer you are managing?

1 – Not at all satisfied

2 – Somewhat dissatisfied

3 – Neither satisfied nor dissatisfied

4 – Somewhat satisfied

5 – Completely satisfied

6. On a scale of 1-5, how satisfied are you currently with the level of anatomical detail *DOCUMENTED* following your MDM for CT scans of patients you are treating with pancreas cancer?

1 – Not at all satisfied

2 – Somewhat dissatisfied

3 – Neither satisfied nor dissatisfied

4 – Somewhat satisfied

1. – Completely satisfied

7. Regarding pancreas cancer tumour/vessel relationships based on CT scans as discussed in your MDT meetings, you currently feel you….

- would like more information OR
- are provided sufficient information OR
- are provided too much information

1. Please rate the ease with which you can determine the resectability status of pancreatic tumours, considering the level of anatomical detail (tumor/vessel relationship) DISCUSSED in your MDM based on the CT scans of patients you are treating for pancreatic cancer.

1 – Very difficult

2 – Difficult

3 – Neither difficult nor easy

4 – Easy

5 – Very easy

9. Do you think synoptic/structured reporting of pancreas cancer CT scans will be useful?

- Yes
- No
- Maybe

- Unsure / Don’t know

1. If you work in more than one institution, we encourage you to comment on any differences in approach to determining the resectability status of pancreatic tumors between them. (free text)

11. Any final comments please? (free text)

**Below are the questions that will appear if the respondent is a radiologist:**

5. Have you used synoptic CT scan reporting in any tumour types before?

- Yes, regularly
- Yes, occasionally
- No
- Unsure / Don’t know
  1. And if the answer is Yes, which tumour types? (free text)

6. Do you already use a Template report for non-metastatic PDAC?

- Yes
- Sometimes
- No
- Unsure / Don’t know

7. Do you routinely include a resectability status in your reporting of PDAC?

- Yes/Always
- Sometimes
- No

8. On a scale of 1-5, how satisfied are you with the level of anatomical detail discussed in your MDT for CT scans of patients with PDAC?

1 – Not at all satisfied

2 – Somewhat dissatisfied

3 – Neither satisfied nor dissatisfied

4 – Somewhat satisfied

1. – Completely satisfied

9. Do you think synoptic/structured reporting of PDAC will be useful?

- Yes
- No
- Maybe
- Unsure / Don’t know

10. Do you think synoptic reporting will enhance your efficiency and accuracy of reporting?

- Yes
- No
- Maybe
- Unsure / Don’t know

11. If you work in more than one institution, we encourage you to comment on any differences in approach to determining the resectability status of pancreatic tumors between them. (free text)

12. Any final comments please? (free text)

**SCANPatient Clinician Satisfaction Survey – 6 months after the use of synoptic report**

1. How satisfied are you with the level of anatomical detail *DISCUSSED* in your MDT now that you are using the synoptic report for CT scans of patients with pancreas cancer (compared with prior to using the synoptic report)?

0 5 10

Much worse About the same Much better

than before as previously than before

1. How satisfied are you with the level of anatomical detail *DOCUMENTED* following your MDT for CT scans of patients since the introduction of the synoptic report?

0 5 10

Much worse About the same Much better

than before as previously than before

If you are a surgeon, please continue to answer Questions #3 to #6 below:

If you are a radiologist, please directly go to answer Question #7 to #11 below:

1. Regarding pancreas cancer tumour/vessel relationships as discussed in your MDT meetings, you currently feel you:

- would like more information
- are provided sufficient information
- are provided too much information

in relation to the relevant arteries and veins.

1. How have you found using the synoptic report?

- Highly valuable
- Moderately valuable
- Of minimal interest
- Purely academic and for novelty value only

1. Do you think scan interpretation and resectability conclusion has changed since the introduction of the synoptic report, with particular reference to the borderline resectable category?

- More patients considered borderline
- Fewer patients considered borderline
- The same number of patients as previously considered borderline

1. Any final comments please? (free text)

(Questions #7 to #11 below are for radiologists only)

1. On a scale of 1-10, how satisfied are you with the amount of detail required for the synoptic report?

0 1 2 3 4 5 6 7 8 9 10

Not at all satisfied completely satisfied

1. Do you find the synoptic template easy to use?

- Yes
- Sometimes
- No

1. Do you believe the template increases your accuracy of reporting of PDAC?

- Yes
- Sometimes
- No

1. Do you believe the template is more time-efficient?

- Yes
- Sometimes
- No

1. Any final comments please? (free text)

Appendix 2. Data management plan – identifiable data

SCANPatient Data Management Plan

**PRIMARY DATA SOURCES**

**During the standard reporting phase**, the nominated radiologists will not provide any intervention to their current practices. During this period, the authorised central operation team staff, will collect the **MDM (Multidisciplinary Meeting)** **discussion report** from sites and identify patients who have **suspected, proven, confirmed and recurrent pancreatic ductal adenocarcinoma (PDAC)**. This approach aims to alleviate the site workload, as some **site liaisons** are doctors who have limited time for administrative tasks. Alternatively, the site liaisons can provide the central team with a specific MDM list, containing only the identified PDAC patients. Those patients will be included in the study, and the central team will request certain reports for them such as the **radiology report**, biopsy report, the **operation** and **pathology reports**, if patients undergo surgery.

Most PDAC patients will already have metastasis at the time of diagnosis (and may not even be presented at the MDM, eliminating them from data collection), thus many of the patients included in the study will not have surgery. This means that only the radiology report will be collected for these patients.

**During the synoptic reporting phase**, the nominated radiologists will identify PDAC patients from the MDM list and fill out the **synoptic report form** in the **SCANPatient REDCap database**. REDCap is a secure and robust web-based application that is specifically designed for the construction and management of research databases. This platform is hosted locally in Australia by Helix, a Monash Technology Research Platform dedicated to supporting research involving sensitive data, such as personal or health-related information. It is also designed to function as a secure file transfer platform, facilitating the upload and download of documents. It is a university-wide capability, comprising of expert staff and fortified by world-class research infrastructure. REDCap requires two-factor authentication for access and is only accessible to permitted users. Users outside of Monash can only be granted access by the authorised central team staff*.*

After filling out the synoptic report form, the radiologist can generate and print a PDF version of the report, which can be used as a reference during the MDM. **It is essential to emphasise that the synoptic report is NOT an official interpretation of the CT scan, but rather functions as a document specifically to facilitate the MDM discussion.** It is also important to note that the data entered by the radiologists at each site are only visible to the database users at that particular site, thus ensuring confidentiality and privacy.

**During the synoptic phase, the site liaisons will continue to send the radiology, biopsy, operation, and pathology reports to the central team.**

**DATA TRANSFER AND STORAGE**

The authorised central team staff will create a list of the pending reports per site and **password-protect the document**. This password-protected document will be sent out to sites. The site liaisons will transmit the requested patient reports via **REDCap** (previously described) or **Monash Drive (MDrive). MDrive** is a secure platform developed by IBM, and is hosted at Monash University. The Monash Drive operates on a hybrid-cloud infrastructure, with the interface being hosted on IBM Aspera On Cloud. The data is stored and managed within Monash On-Premise Storage using Secure Data Enclaves, which is certified under Monash ISO 27001 Certified Infrastructure. The connection to MDrive is a point-to-point connection, ensuring end-to-end encryption. This means that the data remains secure and does not pass through any third-party servers during transmission to or from the storage.

If the above-mentioned process is not feasible, the following methods will be employed:

- In cases where sites do not have the capacity to send the requested reports, the authorised central team staff will request direct access to the sites' patient information system/electronic medical records, subject to approval. This access may be either remote or on-site.
- If technical or other issues prevent sites from using either MDrive or REDCap for report transmission, sites can send the reports via email, provided that each file is password-protected.
- For sites encountering difficulties in sending reports electronically, hard drives, USB sticks, or any other robust storage devices will be used to collect the reports, with the requirement that the files are password-protected. Passwords will be sent via secure messaging methods.
- In situations where sites need to send detailed requisite data rather than raw reports, they may choose to do so if they have the capacity.
- If none of the above methods are suitable for certain sites, those sites will need to designate a data entry person to collect the study data from the reports and enter them directly into the database. Training will be provided by the authorised central team staff.

After the requested reports or data are sent, uploaded or collected, the authorised central team staff will save them in the **SCANPatient folder** in the Monash S: Drive. **S: Drive** is a secure Monash network storage designated for storing all types of research data, including very sensitive, sensitive, restricted, and public data. Access to the S: Drive is limited to Monash staff and can only be accessed through the Monash VPN (Virtual Private Network). Moreover, access to the Monash Network requires the use of the OKTA authenticator, which is linked to each user’s mobile.

The SCANPatient folder within the S: Drive is only accessible to the authorised central team staff. Once the documents have been downloaded and saved in the S: Drive, the existing documents in the MDrive, REDCap, secure emails, password-protected hard drives and USB sticks or other password protected storage devices will be deleted.

To ensure the seamless management of data flow at the central site, it is important to have a minimum number of individuals (only authorised central team staff) will have access to the identified data. This redundancy helps maintain consistency and reduces the risk of disruptions or delays in data handling.

**It is crucial to note that the central investigators** **(in contrast to the employed project staff all of whom have been trained in secure processes) will not have access to identifiable datasets/medical reports transmitted by sites.** This safeguard ensures the privacy and confidentiality of the data and maintains compliance with ethical and regulatory standards.

**DATA EXTRACTION**

The authorised central team staff will be responsible for data extraction and entry throughout the standard and synoptic reporting phases, while the nominated radiologists or their designated clinician staff will actively participate in data entry during the synoptic phase by providing their descriptions of the CT images in a structured manner in the REDCap database or on a paper-based synoptic form. In instances where reports are not authorised to be sent to the central team, the designated site data collector will directly enter the required data into the SCANPatient REDCap database. Training will be provided to the site data collector(s). In instances where sites need to send detailed requisite data rather than the raw reports, they have the option to do so if they have the capacity.

**DATA LINKAGE**

Towards the end of the study, the authorised central team staff or designated site-based staff will apply for information regarding death status from each site or establish a process to link demographic data to the national death registry to obtain the death data. Such linkages require that study datasets that are submitted contain certain information, such as **surname, given names, sex, and date of birth**, to facilitate an accurate linkage process. Other possible death checks will also be explored in order to get the latest death data available at the end of the data collection period. If the authorised central team staff have access to sites’ patient information system/electronic medical records, death data will be collected from the patient record system (where these data are available). The site liaisons or the designated data collectors may also collect the death data from their patient information system/electronic medical records or from any available site death data sources. Site-based death data collection can be done on a regular basis.

The death data are essential to the calculation of the overall survival rate of patients.

**DATA RETENTION**

The data entered into REDCap by the radiologists and/or the authorised central and site-based staff will be stored in REDCap during the entire data collection period.

Once data collection ceases, the authorised central team staff will take an additional security measure by password-protecting the folders in the S: Drive. All the data and files saved in the S: Drive will be retained in the same folder or in the Monash Secure Data Enclaves for a period of 15 years after publication, in compliance with Monash University's Retention and Disposal Authority. This compliance is based on the standards outlined by the Public Record Office Victoria Disposal Standards. By adhering to these retention guidelines, the study data will be preserved for long-term access and potential future reference.

**DATA ANALYSIS**

During the data collection period, the authorised central team staff will export data from REDCap into an EXCEL spreadsheet or other statistical package software on a regular basis and perform basic data analysis and cleaning. The data will be used to provide update on the progress of data collection. All data analyses files will be stored securely in S: Drive.At the end of the study, the authorised central team staff will perform a final export of the entire dataset from REDCap to an EXCEL spreadsheet or other statistical package software. This comprehensive dataset will undergo final data validation checks and data cleaning processes to ensure its accuracy and integrity.

Following the validation and cleaning steps, the authorised central team staff will proceed to de-identify the dataset. Once de-identified, the dataset will be transmitted to the Monash University study statistician via secure methods for further analysis and interpretation. The statistician will utilise the de-identified dataset to conduct in-depth statistical analysis and assist in the interpretation of the study findings

**DATA SECURITY**

To ensure compliance with ethical guidelines and data protection standards, all authorised personnel at the central site who handle identifiable data, have received training in ICH-GCP (International Council for Harmonisation of Technical Requirements for Pharmaceuticals for Human Use - Good Clinical Practice) and Data Privacy and Confidentiality trainings. These training programs equip personnel with the necessary knowledge and understanding of their obligations outlined in the National Statement and the Australian Code for the Responsible Conduct of Research 2018 (Australian Code). On the other hand, Monash University places a strong emphasis on protecting the privacy of individuals' personal and health information. The university adheres to relevant privacy laws, including the Privacy and Data Protection Act 2014 (Vic) and the Health Records Act 2001 (Vic), as well as other applicable privacy regulations.

In the interest of maintaining a secure environment, any suspicious activity or potential cyber security incidents, whether they involve intentional or accidental occurrences, will be promptly reported to **eSolutions team,** Monash University's IT support staff. They will be reached through their hotline **(0399051777)** or through their dedicated website at [myServices - Home (onbmc.com)](mailto:myServices%20-%20Home%20(onbmc.com)) or by contacting the **cyberteam@monash.edu** email address. In cases where personal information is involved, the incident will also be reported to Monash University's Data Protection and Privacy Office.

In the event of a cyber security incident, Monash University follows its established University Cyber Incident Response process. This process ensures compliance with applicable legal requirements, mitigates harm to affected individuals, and minimises damage and risk to the university. The university conducts regular security testing to assess vulnerabilities across systems, processes, and personnel. The results of these tests inform ongoing improvements to the management of cyber risks and the implementation of controls to prevent cyber threats.

The University implements and maintains an ISO/IEC 27001-certified Information Security Management System (ISMS) for a specific scope of systems and their underlying infrastructure and operational processes, which are defined within the ISMS scope.

**DATA DISPOSAL**

The SCANPatient dataset will be retained and disposed of in compliance with Monash University's Retention and Disposal Authority, which is based on the Public Record Office Victoria Disposal Standards relevant to the University. The secure folders that will be used to store the dataset and medical reports will be disposed of as soon as possible, 15 years after publication, to ensure compliance with the Privacy and Data Protection Act. To initiate the process of records destruction, an Application for Destruction of Records will be completed and authorised by the Monash Professor of Cancer Research. The completed form will be submitted to the Monash Archives Manager, who will either approve or reject the application and provide guidance as needed. If the application is approved, the SCANPatient researchers will coordinate with eSolutions team to securely destroy the records. All electronic records in REDCap will be destroyed by **Helix Administrators**, REDCap host in Monash University and all existing copies of the records saved in Monash's S: drive or in the Monash Secure Data Enclaves and MDrive will be identified and destroyed, including system backups.

By following this process, Monash University ensures the proper disposal of the SCANPatient dataset and medical reports, maintaining compliance with relevant regulations and safeguarding the privacy and confidentiality of the data.

**NOTE: The data management plan may undergo minor changes from time to time.  If proposed changes impact the existing approvals by the MH HREC or RGO, the relevant ethics and governance offices will be notified and relevant processes followed.**

Appendix 3. Data management plan – de-identifiable data

SCANPatient Data Management Plan

**PRIMARY DATA SOURCES**

**During the standard reporting phase**, the nominated radiologists will not provide any intervention to their current practices. During this period, the site liaisons will identify the **suspected, proven, confirmed and recurrent pancreatic ductal adenocarcinoma (PDAC)** from the MDM outcome list and transmit to the central site a redacted MDM outcome report containing only the identified PDAC patients. Most PDAC patients will already have metastasis at the time of diagnosis (and may not even be presented at the MDM, eliminating them from data collection), thus most of the patients included in the study will not have surgery. This means that only the **redacted radiology, biopsy and MDM outcome reports** will be collected for these patients. The redacted **operation and pathology reports** will also be requested if patients undergo surgery.

**During the synoptic reporting phase**, the nominated radiologists will identify PDAC patients from the MDM list. Since the patients’ identifiers will not be provided by sites, the reporting radiologists and the site liaisons will work together to assign a patient code to each identified PDAC patient. This code must be recorded in the patient log (see description below), along with the patients’ identifiers. The patient codes can be assigned in sequential order to facilitate easier code assignment. After a patient has been assigned a patient code, the nominated radiologist will proceed to complete a **synoptic report form** in the SCANPatient REDCap database. **REDCap** is a secure and robust web-based application that is specifically designed for the construction and management of research databases. This platform is hosted locally in Australia by Helix, a Monash Technology Research Platform dedicated to supporting research involving sensitive data, such as personal or health-related information. It is also designed to function as a secure file transfer platform, facilitating the upload and download of documents. It is a university-wide capability, comprising of expert staff and fortified by world-class research infrastructure. REDCap requires two-factor authentication for access and is only accessible to permitted users. Users outside of Monash can only be granted access by the authorised central team staff from the central team*.* After filling out the synoptic report form, the radiologist can generate and print a PDF version of the report, which can be used as a reference during the MDM. **It is essential to emphasise that** **the synoptic report is NOT an official interpretation of the CT scan, but rather functions as a document specifically to facilitate the MDM discussion.** It is also important to note that the data entered by the radiologists at each site are only visible to the database users at that particular site, thus ensuring confidentiality and privacy.

**During the synoptic phase, the site liaisons will continue to send the radiology, biopsy, operation, and pathology reports to the central team.**

**PATIENT LOG**

It is imperative that the site liaisons maintain a **patient log** to ensure that the de-identified records in the database have been assigned patient codes that are linked correctly to their respective patient identifiers. The patient log will be managed and maintained using an EXCEL spreadsheet, which must be password-protected for added security. The file should only be stored within the site’s secure network and folder.

Below is a sample patient log in EXCEL spreadsheet:


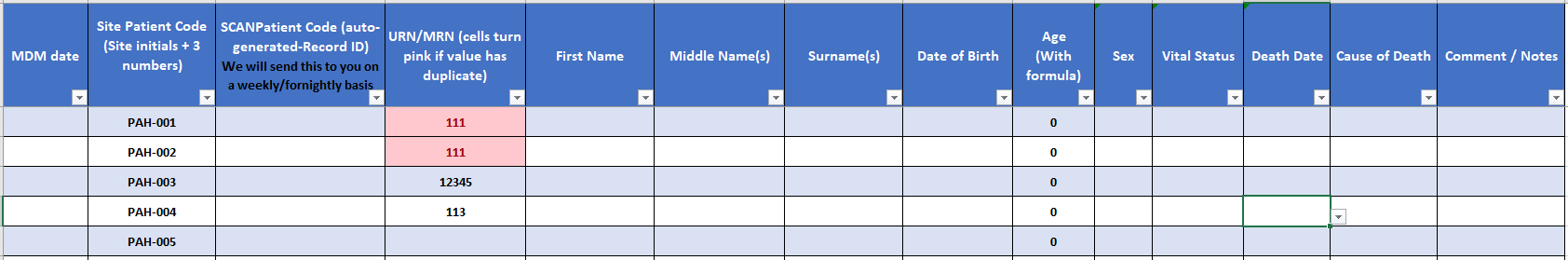


The patient log must include at least the following information:

1. **MDM date**
2. **Site Patient code** (format: 3-char. hospital code + hyphen + 3-digit number) e.g. MOH-001. This will be assigned by either the reporting radiologist or the site liaison, depending on their agreement.
3. **URN/MRN**
4. **First Name**
5. **Middle Name(s) (if applicable)**
6. **Last Name(s)**
7. **Date of birth**
8. **Age at enrolment (calculated field)**
9. **Sex**
10. **Vital status**
11. **Death date**
12. **Cause of Death**
13. **SCANPatient code** (Record ID in REDCap) See below for reference.


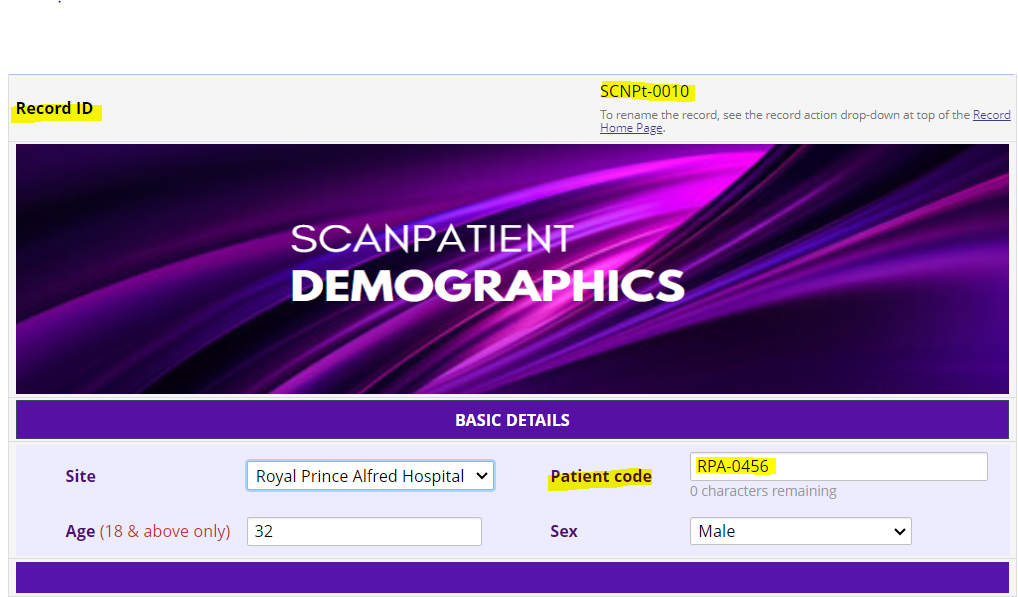


**During the standard reporting phase**, the central team will send the SCANPatient codes, along with their corresponding patient codes to the sites on a monthly basis. The site liaisons will match these codes with their patient logs to maintain accurate patient identification and proper linkage of patient information to the records in the database.


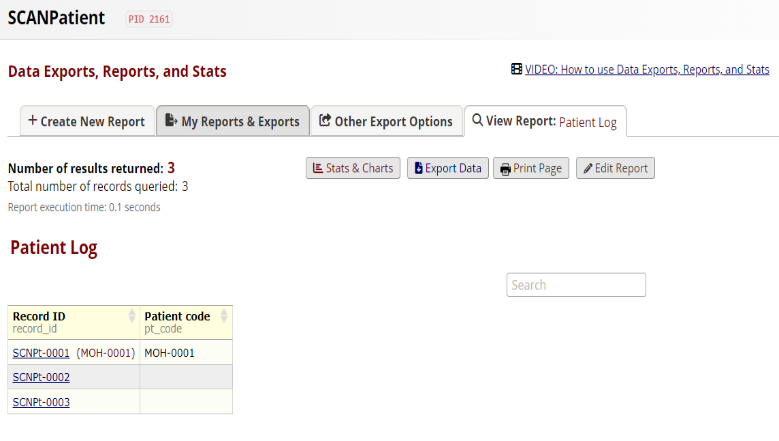
**During the synoptic reporting phase**, the site liaisons will have to extract the SCANPatient code from REDCap and copy and match these codes to their corresponding patient identifiers in the patient log. Alternatively, the site liaisons can export a custom report in REDCap called “**Patient Log**” into an EXCEL spreadsheet so that the matching of patients’ information with their corresponding SCANPatient codes can be done effortlessly. See below for a sample of a patient log report in REDCap. Training can be provided to implement this process if needed.

**Please keep in mind that the actual implementation and workflow may vary based on the specific needs and dynamics at each site.**

**REPORT REDACTION**

The site liaisons will redact or censor identifiers on the reports prior to sending them to the central site. This can be done by cropping the document or shading the identifiers to render them unreadable. The age and sex information must remain uncensored on the document. The patient code, which will serve as the unique identifier, must be written on the document. Below is a sample of a redacted report with a patient code.

**Age and Sex**


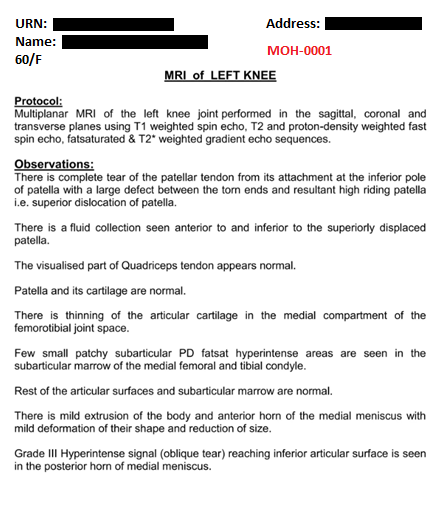


**Patient Code**

**DATA TRANSFER AND STORAGE**

The authorised central team staff will create a list of the pending reports per site and **password-protect the document**. This password-protected document will be sent out to sites. The site liaisons will transmit the requested patient reports via **REDCap** (previously described) or **Monash Drive (MDrive). MDrive** is a secure platform developed by IBM, and is hosted at Monash University. The Monash Drive operates on a hybrid-cloud infrastructure, with the interface being hosted on IBM Aspera On Cloud. The data is stored and managed within Monash On-Premise Storage using Secure Data Enclaves, which is certified under Monash ISO 27001 Certified Infrastructure. The connection to MDrive is a point-to-point connection, ensuring end-to-end encryption. This means that the data remains secure and does not pass through any third-party servers during transmission to or from the storage.

If the above-mentioned process is not feasible, the following methods will be employed:

- If technical or other issues prevent sites from using either MDrive or REDCap for report transmission, sites can send the reports via email, provided that all identifiable data have been redacted.
- For sites encountering difficulties in sending reports electronically, hard drives, USB sticks, or any other robust storage devices will be used to collect the reports, provided that all identifiable data have been redacted.
- In situations where sites need to send detailed requisite data rather than raw reports, they may choose to do so if they have the capacity.
- If none of the above methods are suitable for certain sites, those sites must designate a data entry person to collect the study data from the reports and enter them directly into the database. Training will be provided by the authorised central team staff.

After the requested reports or data are sent, uploaded or collected, the authorised central team staff will save them in the **SCANPatient folder** in the Monash S: Drive. **S: Drive** is a secure Monash network storage designated for storing all types of research data, including very sensitive, sensitive, restricted, and public data. Access to the S: Drive is limited to Monash staff and can only be accessed through the Monash VPN (Virtual Private Network). Moreover, access to the Monash Network requires the use of the OKTA authenticator, which is linked to each user’s mobile.

The SCANPatient folder within the S: Drive is only accessible to the authorised central team staff, and the senior research officer. Once the documents have been downloaded and saved in the S: drive, the existing documents in the MDrive, REDCap, secure emails, password-protected hard drives, USB sticks or other password-protected storage devices will be deleted.

To ensure the seamless management of data flow at the central site, it is important to have a minimum number of individuals (only the authorised central team staff) who will have access to or capable of overseeing the data management process. This redundancy helps maintain consistency and reduces the risk of disruptions or delays in data handling.

**It is crucial to note that the central investigators (in contrast to the employed project staff all of whom have been trained in secure processes) will not have access to identifiable datasets/medical reports transmitted by sites.** This safeguard ensures the privacy and confidentiality of the data and maintains compliance with ethical and regulatory standards.

**DATA EXTRACTION**

The authorised central team staff will be responsible for data extraction and entry throughout the standard and synoptic reporting phases, while the nominated radiologists or their designated clinician staff will actively participate in data entry during the synoptic phase by providing their descriptions of the CT images in a structured manner in the REDCap database or on a paper-based synoptic form. In instances where reports are not authorised to be sent to the central team, the designated site data collector will directly enter the required data into the SCANPatient REDCap database. Training will be provided to the site data collector(s). In instances where sites need to send detailed requisite data rather than the raw reports, they have the option to do so if they have the capacity.

**DATA LINKAGE**

Towards the end of the study, the authorised central team staff or designated site staff will apply for information regarding death status from each site or establish a process to link demographic data to the national death registry to obtain the death data. Such linkages require that study datasets that are submitted contain certain information, such as **surname, given names, sex, and date of birth**, to facilitate an accurate linkage process. For patients whose identifiers are not available, the sites will be requested to obtain the individual vital/death status of patients through their hospital medical record/information system or other death data sources available at the site. Other possible death checks will also be explored in order to get the latest death data available at the end of the data collection period. The site liaisons or the designated data collectors may also collect the death data from their patient record system (where these data are available) or from any available site death data sources. Site-based death data collection can be done on a regular basis.

The death data are essential to the calculation of the overall survival rate of patients.

**DATA RETENTION**

The data entered into REDCap by the radiologists and/or the authorised central and site-based staff will be stored in REDCap during the entire data collection period.

Once data collection ceases, the authorised central team staff will take an additional security measure by password-protecting the folders in the S: drive. All the data and files saved in the S: drive will be retained in the same folder or in the Monash Secure Data Enclaves for a period of 15 years after publication, in compliance with Monash University's Retention and Disposal Authority. This compliance is based on the standards outlined by the Public Record Office Victoria Disposal Standards. By adhering to these retention guidelines, the study data will be preserved for long-term access and potential future reference.

**DATA ANALYSIS**

During the data collection period, the authorised central team staff will export data from REDCap into an EXCEL spreadsheet or other statistical package software on a regular basis and perform basic data analysis and cleaning. The data will be used to provide update on the progress of data collection. All data analyses files will be stored securely in Monash S: Drive or the Monash Secure Data Enclaves.

At the end of the study, the authorised central team staff will perform a final export of the entire dataset from REDCap to an EXCEL spreadsheet or other statistical package software. This comprehensive dataset will undergo final data validation checks and data cleaning processes to ensure its accuracy and integrity.

Following the validation and cleaning steps, the authorised central team staff will proceed to de-identify the dataset. Once de-identified, the dataset will be transmitted to the Monash University study statistician via secure methods for further analysis and interpretation. The statistician will utilise the de-identified dataset to conduct in-depth statistical analysis, and assist in the interpretation of the study findings.

**DATA SECURITY**

To ensure compliance with ethical guidelines and data protection standards, all authorised personnel at the central site who handle identifiable data have received training in ICH-GCP (International Council for Harmonisation of Technical Requirements for Pharmaceuticals for Human Use - Good Clinical Practice) and Data Privacy and Confidentiality trainings. These training programs equip personnel with the necessary knowledge and understanding of their obligations outlined in the National Statement and the Australian Code for the Responsible Conduct of Research 2018 (Australian Code). On the other hand, Monash University places a strong emphasis on protecting the privacy of individuals' personal and health information. The university adheres to relevant privacy laws, including the Privacy and Data Protection Act 2014 (Vic) and the Health Records Act 2001 (Vic), as well as other applicable privacy regulations.

In the interest of maintaining a secure environment, any suspicious activity or potential cyber security incidents, whether they involve intentional or accidental occurrences, will be promptly reported to **eSolutions team,** Monash University's IT support staff. They will be reached through their hotline **(0399051777)** or through their dedicated website at [myServices - Home (onbmc.com)](mailto:myServices%20-%20Home%20(onbmc.com)) or by contacting the **cyberteam@monash.edu** email address. In cases where personal information is involved, the incident will also be reported to Monash University's Data Protection and Privacy Office.

In the event of a cyber security incident, Monash University follows its established University Cyber Incident Response process. This process ensures compliance with applicable legal requirements, mitigates harm to affected individuals, and minimises damage and risk to the university. The university conducts regular security testing to assess vulnerabilities across systems, processes, and personnel. The results of these tests inform ongoing improvements to the management of cyber risks and the implementation of controls to prevent cyber threats.

The University implements and maintains an ISO/IEC 27001-certified Information Security Management System (ISMS) for a specific scope of systems and their underlying infrastructure and operational processes, which are defined within the ISMS scope.

**DATA DISPOSAL**

The SCANPatient dataset will be retained and disposed of in compliance with Monash University's Retention and Disposal Authority, which is based on the Public Record Office Victoria Disposal Standards relevant to the University. The secure folders that will be used to store the dataset and medical reports will be disposed of as soon as possible, 15 years after publication, to ensure compliance with the Privacy and Data Protection Act. To initiate the process of records destruction, an Application for Destruction of Records will be completed and authorised by the Monash Professor of Cancer Research. The completed form will be submitted to the Monash Archives Manager, who will either approve or reject the application and provide guidance as needed. If the application is approved, the SCANPatient researchers will coordinate with eSolutions team to securely destroy the records. All electronic records in REDCap will be destroyed by **Helix Administrators**, REDCap host in Monash University and all existing copies of the records saved in Monash's S: drive or Monash Secure Data Enclaves and MDrive will be identified and destroyed, including system backups.

By following this process, Monash University ensures the proper disposal of the SCANPatient dataset and medical reports, maintaining compliance with relevant regulations and safeguarding the privacy and confidentiality of the data.

**NOTE: The data management plan may undergo minor changes from time to time.  If proposed changes impact the existing approvals by the MH HREC or RGO, the relevant ethics and governance offices will be notified and relevant processes followed.**
